# Effects of Malic Acid Vase Solution on the Postharvest Quality and Vase Life of Cut Rose ‘Candlelight’ Grown Under Low-Light Conditions

**DOI:** 10.3390/plants15203079

**Published:** 2026-10-09

**Authors:** Ji Na Kim, Young Boon Lee

**Affiliations:** Department of Horticulture, The Chonnam National University, Gwangju 61186, Republic of Korea; jjinaz7728@gmail.com

**Keywords:** carbohydrate, climate-resilient, cut flower, petal color, postharvest physiology, preservative, *Rosa hybrida* L., vase solution uptake, water relations

## Abstract

Low-light conditions during winter cultivation reduce the postharvest quality of cut roses, highlighting the importance of effective postharvest management. This study investigated the effects of malic acid (MA) and sucrose (Su) vase solutions on the postharvest quality and vase life of cut rose (*Rosa hybrida* L.) ‘Candlelight’ grown under low-light conditions. Cut flowers were maintained in distilled water (DW), tap water (TW), 1% chrysal (CR), 50 mg·L^−1^ malic acid (MA), 1% sucrose (Su), or 50 mg·L^−1^ malic acid + 1% sucrose (MA + Su). MA resulted in the longest vase life, extending it by 22–29% compared with DW and TW, although a significant difference was observed only between MA and DW. It also maintained relatively high vase solution uptake and RFW and prevented basal stem browning. In contrast, Su reduced vase life by 40–43% compared with DW and TW despite increasing leaf carbohydrate content, whereas MA + Su resulted in a significantly shorter vase life than MA alone. Correlation analysis revealed that vase life was positively associated with RFW and flower diameter and negatively associated with non-photochemical quenching (NPQ), fluorescence decline ratio (Rfd), and the petal color parameters L* and C*. These findings suggest that MA treatment sustained vase solution uptake and relative fresh weight, thereby contributing to the maintenance of postharvest quality and prolonged vase life.

## 1. Introduction

The global flower export market was valued at USD 22.4 billion in 2020, with cut flowers accounting for approximately USD 8.7 billion [1]. Roses represented approximately 35% of global cut flower exports by value, with the Netherlands, Ecuador, Kenya, Colombia, and Ethiopia being the major rose-exporting countries [1,2,3]. In Ethiopia, approximately 20% fresh loss in the cut flower industry has been attributed to poor postharvest handling, emphasizing the importance of postharvest management [1]. The postharvest quality and vase life of cut flowers such as roses are influenced by both preharvest and postharvest factors [4,5]. The postharvest quality and vase life of cut flowers are influenced by preharvest factors, such as light, temperature, relative humidity, VPD, irrigation, and nutrient management, as well as harvest and postharvest factors, including harvest stage, pulsing and preservative treatments, storage conditions, transportation environment, and water and microbial management. Roses are high-light-demanding crops that require a daily light integral (DLI) exceeding 30 mol·m^−2^·d^−1^ under greenhouse cultivation, with a minimum of 13 mol·m^−2^·d^−1^. At DLI values below this threshold, normal growth, flowering, and postharvest quality are adversely affected [6,7,8]. In Korea, solar radiation is lowest from December to January [8,9]. Field measurements under commercial greenhouse conditions showed that the average winter (December to February) DLI was 9.4 mol·m^−2^·d^−1^. Low-light conditions during winter have been associated with reduced stem diameter, fewer florets, smaller flower diameter, and shorter vase life in cut roses [10,11]. To mitigate the decline in cut flower quality caused by low-light cultivation, various preharvest cultivation strategies have been investigated, such as the manipulation of light intensity and light quality (e.g., LEDs, high-pressure sodium lamps, red light, and blue light), photoperiod, nocturnal supplemental lighting, and different irrigation regimes [12,13,14].

Vase life is determined by the flower phenotype, which is influenced by the variety, the preharvest cultivation environment, and postharvest factors [5]. Therefore, an integrated approach combining preharvest cultivation and postharvest management is required. Although both preharvest and postharvest periods are major areas of research, postharvest management, also called the second production stage, has received less attention than preharvest management [15]. Numerous studies have focused on improving cut flower quality through preharvest cultivation practices, such as supplemental lighting, CO_2_ fertilization, and nutrient solution management. Additionally, various postharvest technologies, such as vase preservative treatments, have been developed to improve cut flower quality and extend vase life [16,17]. However, relatively few studies have investigated postharvest preservative solutions as a strategy to improve the quality of cut roses produced under low-light conditions [4].

Postharvest studies on vase solutions for cut roses have primarily focused on improving vase life and flower quality through the application of various compounds, including inhibitors of ethylene biosynthesis and action, disinfectants and antimicrobial agents, plant growth regulators, sugars (e.g., sucrose and glucose), organic acids, natural plant extracts, and essential oils [18,19,20,21,22]. Although a wide range of chemical compounds have been used as vase solution additives, the development of compounds that are safe for humans, environmentally friendly, and practically applicable under commercial production conditions is essential for achieving sustainable postharvest management of cut flowers.

Organic acids are synthesized through the incomplete oxidation of photosynthetic products in plants and play important roles in various metabolic pathways, including mitochondrial metabolism, amino acid metabolism, and the glyoxylate cycle [23,24]. Among these organic acids, malic acid is a key intermediate in the tricarboxylic acid (TCA) cycle and is metabolized by malic enzyme in the mitochondria, contributing to ATP production through oxidative phosphorylation [25,26]. Additionally, malic acid plays an important role in maintaining plant physiological homeostasis by regulating vacuolar osmotic balance and intracellular pH [27]. More recently, malic acid has been reported as a promising component of vase solutions for improving the postharvest quality of cut flowers. In cut lisianthus, treatment with 100 mg·L^−1^ malic acid increased water uptake and extended vase life compared with the distilled water control [28]. Similarly, treatment with 150 mg·L^−1^ malic acid alone increased water uptake and prolonged vase life in cut carnation [29]. In cut rose ‘Utopia’, 1 mM malic acid produced a vase life comparable to that achieved with 200 mg·L^−1^ 8-hydroxyquinoline sulfate (8-HQS) [30]. These findings indicate that malic acid has considerable potential as a vase solution additive for improving the postharvest quality of cut flowers.

Sucrose is the most widely used carbohydrate source in vase solutions for cut roses, where it supplements depleted carbohydrate reserves after harvest, thereby maintaining flower opening, respiration, and water balance and ultimately extending vase life [19,31]. In cut roses, sucrose is typically applied in vase solutions at concentrations ranging from 1% to 5%. However, the effects of sucrose applied alone or in combination with antimicrobial agents vary among cultivars [32,33,34,35]. Although malic acid and sucrose have individually been reported to improve postharvest quality, their combined effects in vase solutions have not been comprehensively examined, particularly in cut roses produced under low-light conditions. To address this gap in the literature, this study investigated the effects of vase solutions containing malic acid and sucrose on the postharvest quality and vase life of cut roses produced under low-light conditions during the winter season in Korea.

## 2. Results

### 2.1. Vase Life and Cut Flower Quality

**Figure 1:**
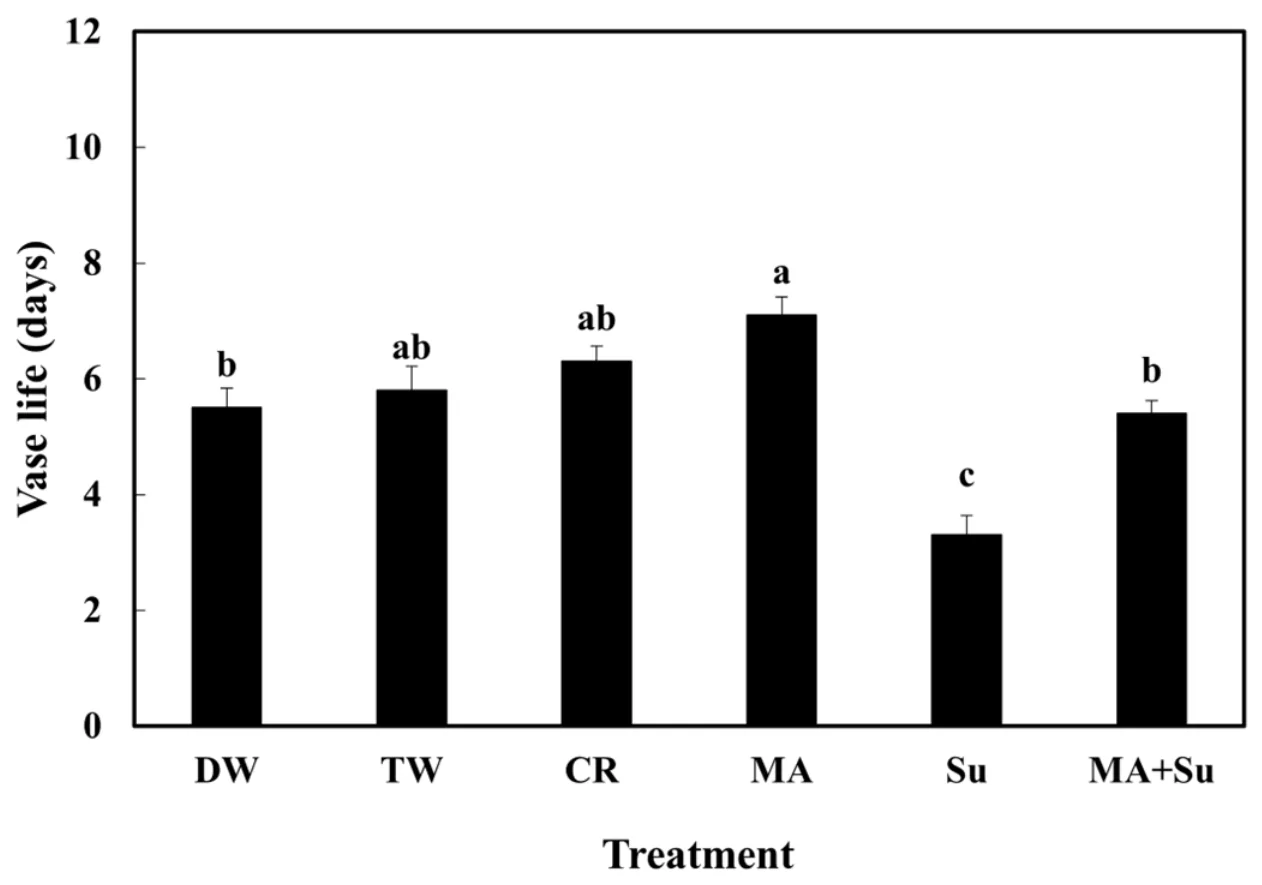
Vase life of the cut rose ‘Candlelight’ treated with different vase solutions. The vase solutions consisted of distilled water (DW), tap water (TW), 1% chrysal (CR), 50 mg·L^−1^ malic acid (MA), 1% sucrose (Su), and 50 mg·L^−1^ malic acid + 1% sucrose (MA + Su). Vertical bars denote standard errors (*n* = 10). Data were analyzed using one-way analysis of variance (ANOVA), followed by Tukey’s honestly significant difference (HSD) post hoc test. Different lowercase letters indicate significant differences among treatments at *p* < 0.05.

**Figure 2:**
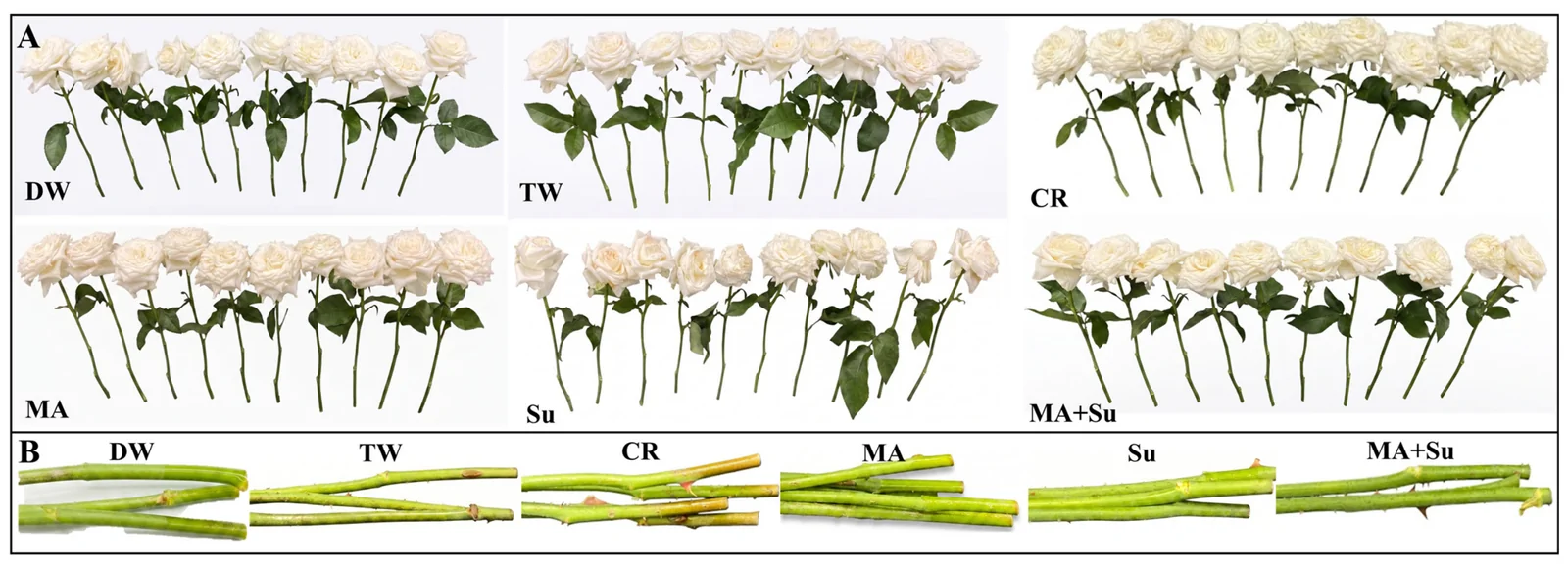
Appearance of cut rose ‘Candlelight’ after 6 days in different vase solutions: (**A**) overall appearance of cut flowers; (**B**) basal stem browning. The vase solutions consisted of distilled water (DW), tap water (TW), 1% chrysal (CR), 50 mg·L^−1^ malic acid (MA), 1% sucrose (Su), and 50 mg·L^−1^ malic acid + 1% sucrose (MA + Su). The photographs are representative images for qualitative comparison and are not shown to scale.

The MA treatment extended vase life by 22–29% compared with the DW and TW treatments; however, a significant difference was observed only between the MA and DW treatments, whereas the difference between the MA and TW treatments was not significant (Figure 1). The Su treatment alone reduced vase life by 40–43% compared with the DW and TW treatments and showed the shortest vase life. The MA + Su treatment had a significantly shorter vase life than the MA treatment, indicating that the addition of sucrose did not improve the vase-life-extending effect of MA. The CR treatment showed only a limited effect on vase life extension and exhibited distinct basal stem browning after 6 days (Figure 2B). In contrast, no basal stem browning was observed following the MA treatment, and flower appearance was better maintained following the DW and TW treatments (Figure 2A). Petal rolling and petal or leaf wilting were the major symptoms associated with vase life termination, although their incidence varied among treatments (Table 1). In the CR treatment, vase life termination was characterized exclusively by petal rolling, with no other symptoms observed. In contrast, the Su treatment resulted in a higher incidence of petal or leaf wilting than of petal rolling.

**Figure 3:**
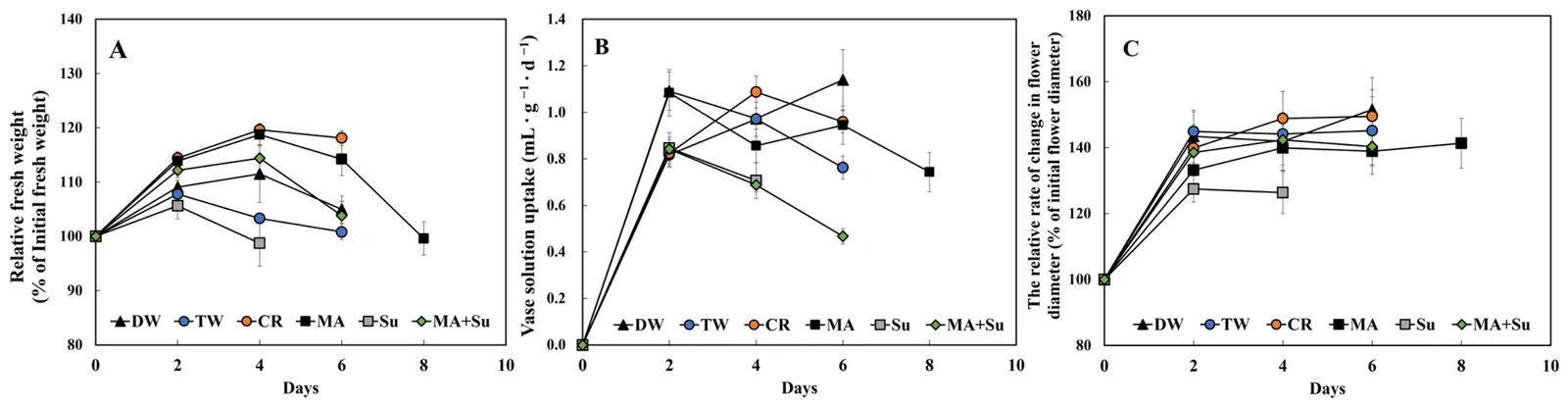
The relative fresh weight (**A**), vase solution uptake (**B**), and relative rate of change in flower diameter (**C**) of the cut rose ‘Candlelight’ treated with different vase solutions. The vase solutions consisted of distilled water (DW), tap water (TW), 1% chrysal (CR), 50 mg·L^−1^ malic acid (MA), 1% sucrose (Su), and 50 mg·L^−1^ malic acid + 1% sucrose (MA + Su). Vertical bars denote standard errors (*n* = 10).

RFW increased during the first 2–4 days after treatment in all vase solution treatments (Figure 3A). The Su treatment was the first to exhibit a decrease below the initial fresh weight (100%) on day 4, whereas the CR and MA treatments maintained RFW above 100% for a longer period (until day 6) than the DW and TW treatments.

Vase solution uptake increased rapidly during the first 2 days in all treatments (Figure 3B). By day 4, corresponding to the average vase life in the Su treatments, both the Su and MA + Su treatments exhibited relatively low vase solution uptake, whereas the CR treatment showed a relatively high value. The MA treatment maintained relatively high vase solution uptake throughout the vase period.

### 2.2. Petal and Leaf Quality

The relative rate of change in flower diameter was lowest in the Su treatment throughout the vase period, although no significant differences were observed among treatments (Figure 3C). The ΔE* was numerically lowest in the Su treatment, followed by the MA treatment, indicating less color change compared to harvest; however, the differences were not statistically significant (Table 2). No significant differences were observed in L or C among treatments. In contrast, h* differed significantly among treatments, with the Su treatment showing a significantly higher value than the MA + Su treatment (Table 2).

**Figure 4:**
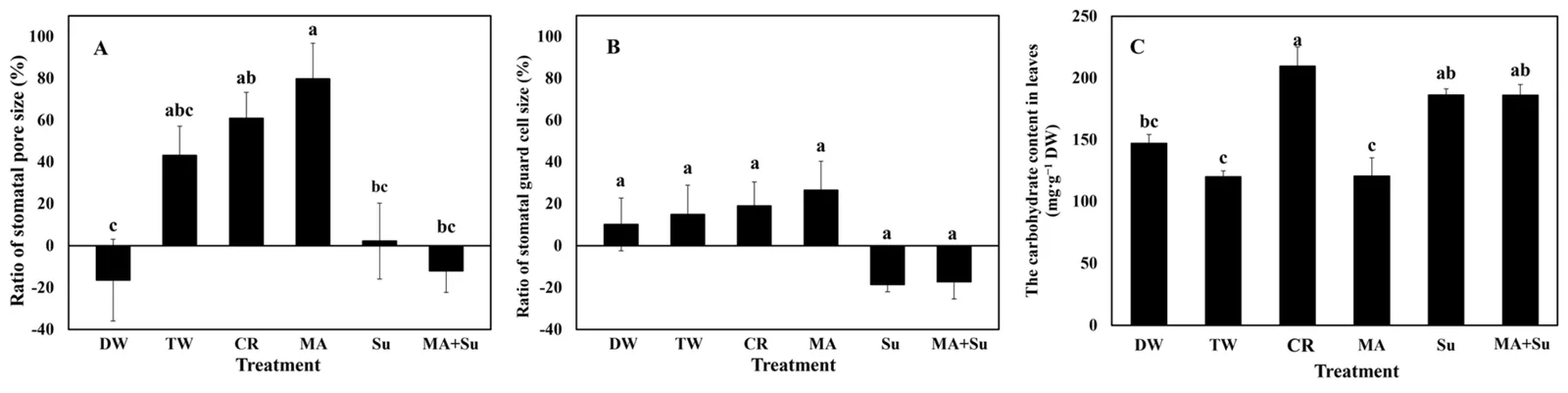
Leaf quality parameters of cut rose ‘Candlelight’ treated with different vase solutions on day 4: (**A**) stomatal pore size; (**B**) guard cell size; (**C**) leaf carbohydrate content. The vase solutions consisted of distilled water (DW), tap water (TW), 1% chrysal (CR), 50 mg·L^−1^ malic acid (MA), 1% sucrose (Su), and 50 mg·L^−1^ malic acid + 1% sucrose (MA + Su). Vertical bars denote standard errors (*n* = 3). Data were analyzed using one-way analysis of variance (ANOVA), followed by Tukey’s honestly significant difference (HSD) post hoc test. Different lowercase letters indicate significant differences among treatments at *p* < 0.05.

To evaluate leaf physiological quality, chlorophyll fluorescence parameters, changes in stomatal pore size, and leaf carbohydrate content were analyzed. No significant differences were observed among treatments in Fv/Fm, NPQ, or Rfd (Table 3). In contrast, QY_Lss and qP were highest in the CR treatment. QY_Lss was significantly higher in the CR treatment than in the TW, MA, Su, and MA + Su treatments but did not differ significantly from that in the DW treatment. The qP in the CR treatment was significantly higher than that in the TW treatment. Although the differences were not statistically significant, the Su treatment exhibited numerically higher NPQ and Rfd values than the other treatments. Leaf carbohydrate content was highest in the CR treatment and remained relatively high in both the Su and MA + Su treatments (Figure 4C). The relative change in stomatal pore size differed significantly among treatments, with the MA treatment showing the highest value, whereas the Su and MA + Su treatments showed relatively low values (Figure 4A). In contrast, no significant differences in guard cell size were observed among treatments, although the MA and CR treatments showed numerically higher values than the other treatments (Figure 4B).

### 2.3. Antimicrobial Effect and Correlation Analysis

**Figure 5:**
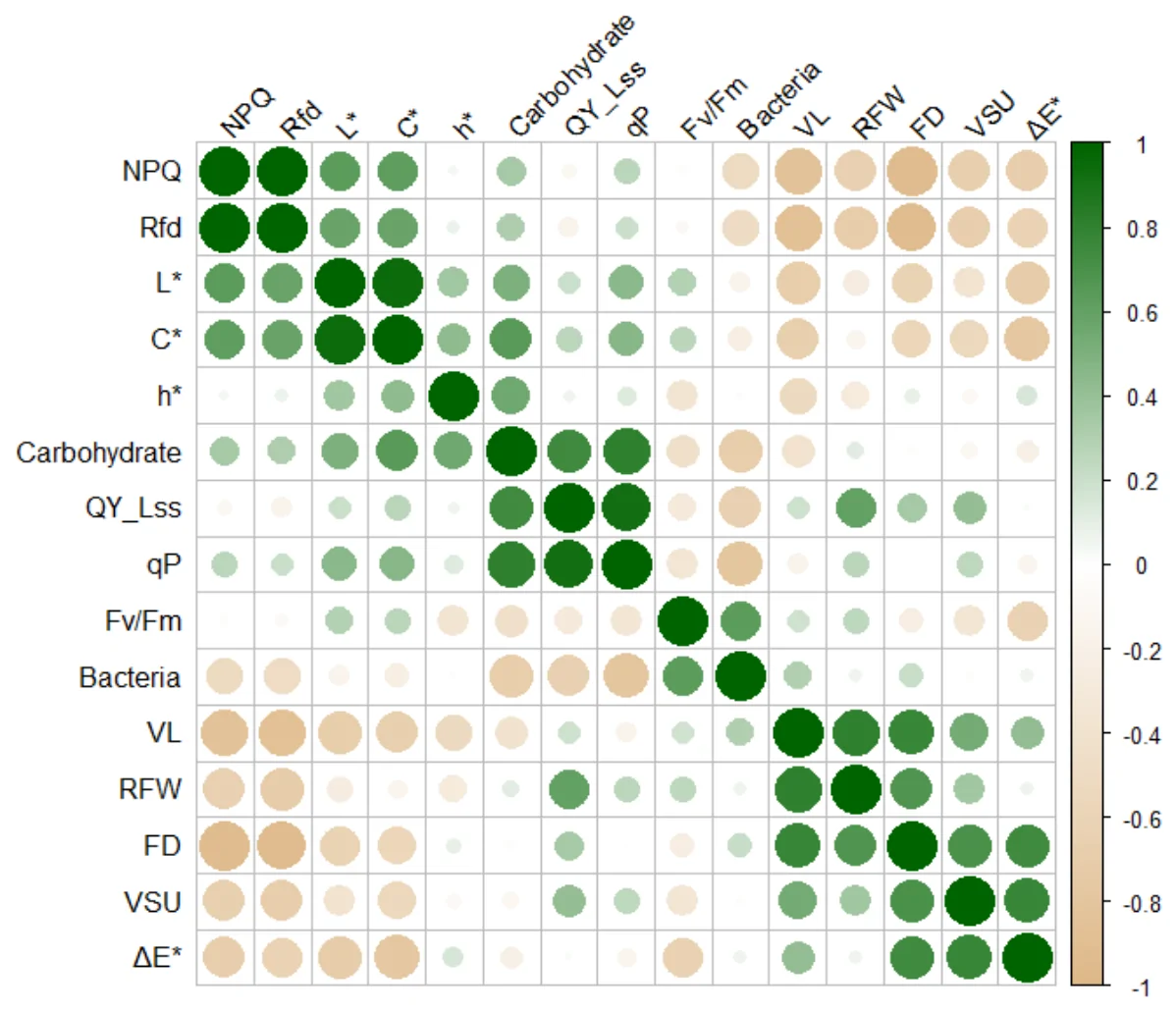
Pearson correlation analysis of the relationships between vase life (VL) and postharvest quality parameters of cut roses. The parameters included non-photochemical quenching (NPQ), steady-state PSII quantum yield (QY_Lss), photochemical quenching coefficient (qP), maximum quantum efficiency of PSII (Fv/Fm), fluorescence decline ratio (Rfd), relative fresh weight (RFW), leaf carbohydrate content, petal color (L*, C*, h*, and ΔE*), bacterial population in the vase solution, flower diameter (FD), and vase solution uptake (VSU). Heatmap of Pearson correlation coefficients (r) for the measured variables, where the color scale indicates positive (green) or negative (orange) correlations and the size of each circle represents the magnitude of the correlation coefficient (|r|), with larger circles indicating stronger correlations. Correlation coefficient values (r = −1.0 to 1.0).

Bacterial populations in the vase solutions were detected at levels of 10^4^–10^5^ CFU·mL^−1^ in all treatments except CR (Table 2), whereas no bacteria were detected in the CR treatment. Bacterial counts in the CR and Su treatments were significantly lower than those in the DW, TW, MA, and MA + Su treatments, whereas no significant difference was observed between the CR and Su treatments. Pearson correlation analysis showed that vase life was strongly negatively correlated with leaf NPQ (*r* = −0.848**), Rfd (*r* = −0.869**), petal L* (*r* = −0.698*), and C* (*r* = −0.667*) regardless of treatment. In contrast, vase life was strongly positively correlated with RFW (*r* = 0.811*) and flower diameter (*r* = 0.780*) (Figure 5).

## 3. Discussion

Senescence symptoms of cut roses vary among cultivars and commonly include petal or leaf wilting, petal rolling, *Botrytis cinerea* infection, flower color changes, and petal or leaf abscission [36]. In the present study, the cut rose cultivar ‘Candlelight’ primarily exhibited petal or leaf wilting and petal rolling as vase life termination symptoms, although their incidence varied among treatments (Table 1). These findings are partly consistent with previously reported senescence symptoms of cut roses and further suggest that the pattern of vase life termination differs among rose cultivars. Therefore, cultivar-specific senescence characteristics should be considered when developing and applying postharvest technologies to extend vase life. The vase life of cut roses typically ranges from approximately 5 to 25 days, depending on the cultivar, although it is relatively short [5]. Therefore, even a one-day extension of vase life can substantially improve marketability during distribution and consumer use [4,5]. In the present study, MA treatment resulted in the longest vase life (7.1 days), extending vase life by 1.3–1.6 days compared with the DW and TW treatments, although a significant difference was observed only between the MA and DW treatments (Figure 1). These findings are consistent with previous studies reporting that malic acid prolonged the vase life of cut *Dianthus* and *Lilium* [28,37]. This suggests that malic acid may have broad applicability as a vase solution additive to extend the vase life of various cut flower species. However, although malic acid showed a significant vase-life-extending effect compared with DW in cut roses produced under low-light conditions in the present study, whether this beneficial effect is specific to the preharvest light environment remains unclear. Cut flower senescence is characterized by physiological changes, including reduced water uptake, depletion of stored carbohydrates, increased respiration, and enhanced ethylene production [38]. Malic acid has been reported to serve as a carbon source, providing metabolic substrates that can partially substitute for stored carbohydrates. Additionally, as an organic anion, malic acid contributes to the maintenance of vacuolar osmotic potential and cell turgor, thereby facilitating water uptake and water balance [39]. Malic acid has also been associated with reduced oxidative damage during cut flower senescence, as indicated by decreased malondialdehyde content and improved membrane stability, thereby contributing to the maintenance of postharvest quality [40]. In contrast, the Su treatment exhibited the shortest vase life (3.3 days), and the combined MA + Su treatment (5.4 days) had a significantly shorter vase life than MA alone. Sucrose is generally regarded as an exogenous carbohydrate source that prolongs vase life by supplementing depleted carbohydrate reserves after harvest [19]. However, although the Su treatment increased leaf carbohydrate content (Figure 4C), vase life was reduced (Figure 1), accompanied by rapid declines in both RFW and vase solution uptake (Figure 3A,B). These findings suggest that, under the conditions of the present study, maintenance of plant water status was more closely associated with vase life than carbohydrate accumulation.

Although many commercial preservatives effectively extend vase life, stem browning is frequently observed, and the development of preservative technologies that extend vase life without inducing stem browning remains an important challenge. In herbaceous cut flowers, however, stem browning may occur as part of the natural senescence process and does not necessarily reduce vase life if stem tissues remain structurally intact [41]. In cut rose ‘Jinny’, chrysal at 0.5 μL·L^−1^ extended vase life, whereas chrysal Professional at 2 mL·L^−1^ did not improve vase life in the cultivar ‘Freedom’ [42,43]. Similarly, in the present study, chrysal (1%) induced basal stem browning in ‘Candlelight’ (Figure 2B) and showed only a limited effect on vase life extension (Figure 1). These findings indicate that the optimal concentration of commercial preservatives varies among rose cultivars, highlighting the need to optimize preservative concentrations or develop alternative vase solution additives that extend vase life without inducing stem browning. In contrast, MA extended vase life without causing stem browning (Figure 1 and Figure 2B), suggesting that malic acid has considerable potential as a vase solution additive for maintaining both vase life and stem quality.

RFW and vase solution uptake in cut roses typically increase during the first 1–2 days after harvest and gradually decline thereafter [44] (Figure 3A,B). This initial increase has been attributed to the recovery of tissue water status through rehydration after transient water deficit induced by harvesting and postharvest handling. Vase life is generally determined as the point at which flowers lose their ornamental value; however, several studies have proposed more objective physiological criteria. For instance, vase life has been defined as the point at which the percentage of cut flower weight loss exceeds a critical threshold [45] or when water balance becomes negative [46]. In the present study, the MA treatment maintained both RFW and vase solution uptake for a longer period than the other treatments (Figure 3A,B), which was consistent with its prolonged vase life. Furthermore, Pearson correlation analysis showed that vase life was strongly positively correlated with RFW (Figure 5), suggesting that maintenance of plant water status is closely associated with vase life extension in cut roses.

In the present study, RFW remained above 100% until day 4 in all treatments except Su, with the CR and MA treatments maintaining the highest values throughout the vase period (Figure 3A). Although no significant differences were observed in vase solution uptake, relatively higher uptake was maintained in the MA and CR treatments, whereas the Su treatment consistently exhibited the lowest uptake (Figure 3B).

Sucrose is widely used as an exogenous carbohydrate source to prolong vase life; however, it may also stimulate microbial proliferation in vase solutions and xylem vessels, leading to vascular occlusion and reduced water uptake [47]. Therefore, sucrose is typically applied in combination with antimicrobial compounds rather than alone for cut roses [48,49]. In the present study, however, bacterial populations in the Su treatment were significantly lower than those in the DW and TW treatments (Table 2), whereas RFW and vase solution uptake declined most rapidly (Figure 3A,B). These findings suggest that the reduction in water uptake observed in the Su treatment cannot be explained solely by bacterial proliferation in the vase solution and that other physiological factors also contributed to the reduced water uptake. Bacterial populations in the MA treatment did not differ significantly from those in the DW or TW treatments (Table 2). In contrast, no bacteria were detected in the CR treatment. These results suggest that the formulation of the commercial preservative may have contributed to suppressing microbial proliferation in the vase solution. Xylem blockage has been reported to occur when bacterial populations in vase solutions reach approximately 10^6^–10^7^ CFU·mL^−1^, resulting in reduced hydraulic conductance and water uptake [50]. In the present study, bacterial populations in the vase solutions remained below the reported threshold, with values around 10^5^ CFU·mL^−1^, suggesting that severe xylem blockage caused solely by bacterial proliferation was unlikely under the experimental conditions. However, bacterial populations may continue to accumulate during commercial distribution and the consumer stage, where flowers are typically held for longer periods. Therefore, further studies evaluating the combined application of malic acid and antimicrobial compounds under extended vase conditions would be valuable [51].

Cut flowers undergo two contrasting physiological phases: flower bud development and opening, followed by maturation, senescence, and wilting. Therefore, postharvest technologies should simultaneously promote flower opening while delaying senescence [38,49]. Sucrose is widely used as an exogenous carbohydrate source to promote flower opening by providing substrates required for cell wall synthesis and respiration during petal expansion [48,49]. In roses, sucrose has been shown to promote flower opening and increase flower diameter [21]. However, in the present study, the Su treatment yielded the smallest flower diameter, although no significant differences were observed among treatments (Figure 3C). Furthermore, the Su treatment resulted in the shortest vase life (Figure 1), indicating that the expected promotive effect of sucrose on flower opening did not occur. These findings are consistent with those of Lee and Kim [31], who reported that sucrose treatment accelerated senescence rather than extending vase life. Collectively, these findings suggest that, under low-light conditions, maintaining plant water status is more important than supplementing exogenous carbohydrates for sustaining flower opening and extending vase life in cut roses.

The Su treatment exhibited the lowest ΔE value, followed by the MA treatment, indicating relatively small changes in petal color compared with harvest; however, no significant differences were observed among treatments (Table 2). Similarly, L and C* values did not differ significantly among treatments. In contrast, h* differed significantly among treatments, with the Su treatment showing a significantly higher value than the MA + Su treatment (Table 2). Lee and Kim [21] reported that sucrose treatment increased L* values while decreasing a* and b* values during vase life in cut roses. However, such responses may vary among rose cultivars with different flower colors and therefore may not be universally applicable. C* is an indicator of color saturation or vividness [52]. Although the Su treatment showed a numerically high C value, C did not differ significantly among treatments (Table 2).

Ha et al. [3] reported that most chlorophyll fluorescence parameters are significantly associated with vase life and can serve as indicators for predicting photosynthetic performance. In the present study, no significant differences were observed among treatments in Fv/Fm, NPQ, or Rfd (Table 3), indicating that the effects of malic acid and sucrose on the maximum photochemical efficiency of PSII and photosynthetic performance were limited. In contrast, QY_Lss and qP were highest in the CR treatment (Table 3), with QY_Lss being significantly higher than in the TW, MA, Su, and MA + Su treatments and qP being significantly higher than in the TW treatment. These results suggest that the CR treatment relatively maintained the efficiency of light energy utilization and the open state of photochemical reaction centers under light-adapted conditions; however, this was not sufficient to extend vase life [53].

Although carbohydrate content is considered an important factor affecting vase life, the present study suggested that water-related traits, including stomatal pore size and vase solution uptake associated with plant water balance, were more closely associated with vase life than leaf carbohydrate content (Figure 4 and Figure 5). Vase life showed the strongest positive correlations with RFW and flower diameter (Figure 5), indicating that maintaining higher RFW and flower diameter is closely associated with prolonged vase life [5]. Additionally, NPQ, Rfd, L*, and C* showed negative correlations with vase life (Figure 5), suggesting that vase life is determined by the combined effects of plant water status and physiological characteristics rather than by a single quality parameter.

## 4. Materials and Methods

### 4.1. Plant Materials, Preparation of Vase Solution, and Treatments

**Figure 6:**
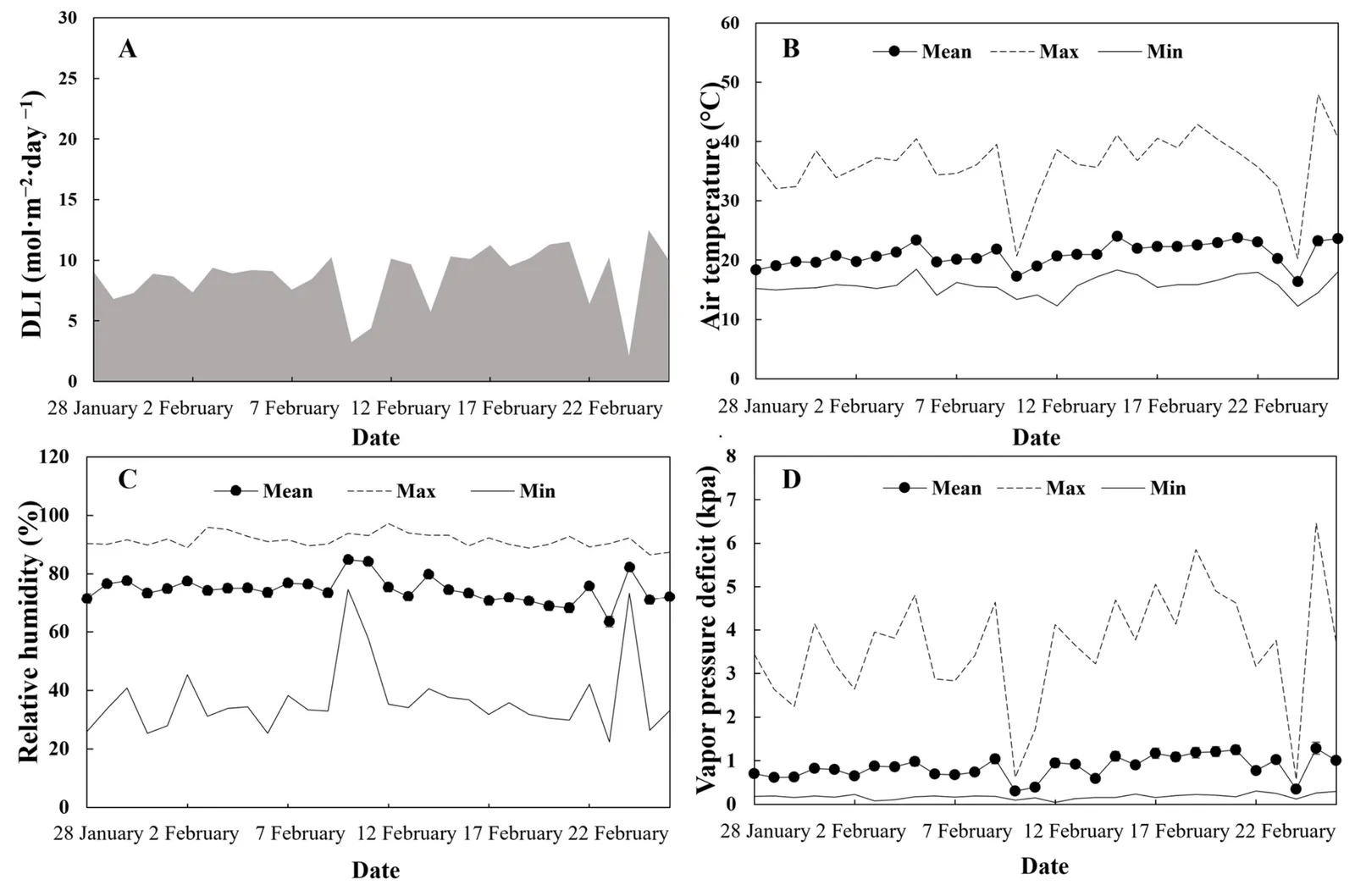
Daily environmental conditions during the month preceding harvest inside the greenhouse in which cut rose ‘Candlelight’ was grown: (**A**) daily light integral (DLI); (**B**) air temperature; (**C**) relative humidity (RH); and (**D**) vapor pressure deficit (VPD). Daily minimum (Min), maximum (Max), and average values (Mean) are presented for each environmental parameter.

The cut rose cultivar ‘Candlelight’ was grown in a hydroponic system and harvested at commercial maturity from a greenhouse in Gwangju, Korea, during the winter from January to February 2026 (Figure 6) [54]. ‘Candlelight’ was selected because it is commercially cultivated year-round at the study site and was therefore suitable for evaluating the postharvest quality of cut roses produced under naturally occurring winter low-light conditions. Daily minimum, maximum, and average light intensity, air temperature, and relative humidity were recorded at hourly intervals using a HOBO data logger (HOBO MX1104, Onset Computer Corporation, Bourne, MA, USA). Hourly mean illuminance (lux) values were converted to photosynthetic photon flux density (PPFD, μmol·m^−2^·s^−1^) using the conversion factor for daylight reported by Thimijan and Heins [55], as follows:

PPFD (μmol·m^−2^·s^−1^) = illuminance (lux)/54.

Daily light integral (DLI, mol·m^−2^·d^−1^) was subsequently calculated from the hourly mean PPFD values as follows:

DLI (mol·m^−2^·d^−1^) = Σ[PPFD_i_ (μmol·m^−2^·s^−1^) × 3600 (s)]/10^6^,

where PPFD_i_ is the hourly mean PPFD for each hour of the day.

Vapor pressure deficit (VPD, kPa) was calculated from air temperature (°C) and relative humidity (%) using the saturation vapor pressure equation described by Allen et al. [56].

The cut stems were transported to the laboratory within 30 min after harvest and recut to 40 cm, and the leaves on the basal 10 cm of the stems were removed [57,58]. The experimental room was maintained under controlled conditions: air temperature of 23 ± 1 °C, relative humidity of 50 ± 5%, and a photosynthetic photon flux density of 15.7 µmol·m^−2^·s^−1^, with a 12-h photoperiod provided by a growth chamber (VS-91G09M-1300, VISION Co., Daejeon, Republic of Korea). Flowers were placed in 500 mL glass bottles containing 300 mL of vase solution. Ten cut flower stems were assigned to each treatment in a completely randomized design. The vase solutions consisted of distilled water (DW), tap water (TW), 1% chrysal (CR, Chrysal International B.V., Naarden, The Netherlands), 50 mg·L^−1^ malic acid (MA, Junsei Chemical Co., Ltd., Tokyo, Japan), 1% sucrose (Su, LPS Solution, Daejeon, Republic of Korea), and 50 mg·L^−1^ malic acid + 1% sucrose (MA + Su). CR was prepared by diluting chrysal to 1% with TW. Malic acid and sucrose were dissolved directly in DW to obtain the designated concentrations. For the MA + Su treatment, both compounds were dissolved together in DW. All treatment solutions were brought to the final volume with DW.

### 4.2. Evaluation of Vase Life and Cut Flower Quality

Vase life was recorded as the number of days following the export simulation until the onset of termination symptoms. These symptoms included petal or leaf wilting (visible wilting affecting ≥50% of the total petal or leaf area), petal and leaf abscission (dropping of one petal or leaf), or peduncle bending (bent neck) with a >30° neck angle [5]. At the end of vase life, petal rolling, petal or leaf wilting, *Botrytis cinerea* symptoms, petal yellowing, petal malformation, and petal or leaf abscission were recorded as vase life termination symptoms. Vase life termination symptoms were expressed as the percentage of cut flowers exhibiting each symptom relative to the total number of cut flowers in each treatment. Because individual cut flowers could exhibit more than one termination symptom, symptoms were recorded independently and the sum of the percentages was not necessarily 100%. Flower color was measured on the outer petals using a color spectrometer (CR-400, Minolta, Japan) based on the CIELAB color space parameters: L* (0, dark; 100, light), a* (red–green; + red; –green), and b* (yellow–blue; + yellow; –blue). C* (chroma) and h* (hue angle) were calculated according to McGuire [52]. Changes in petal color between days 0 and 4 were quantified using the total color difference (ΔE), calculated as ΔE = [(L_4_ − L_0_)^2^ + (a_4_ − a_0_)^2^ + (b_4_ − b_0_)^2^]^1^/^2^.

Relative fresh weight (RFW) and vase solution uptake were determined as described in [59]. RFW was calculated as follows:

RFW (%) = (FW_t_/FW_0_) × 100

where FW_t_ is the fresh weight of the cut flower on day t and FW_0_ is the initial fresh weight on day 0. Vase solution uptake was calculated as follows:

VSU (mL·g^−1^ FW) = (S_t−1_ − S_t_)/FW_0_

where S_t−1_ and S_t_ represent the weights of the vase solution at the previous and current measurement times, respectively, and FW_0_ represents the initial fresh weight of the cut flower.

Flower diameter was measured as the arithmetic mean of the maximum diameter and its perpendicular diameter, measured using digital Vernier calipers (CD-15APX, Mitutoyo Corporation, Kawasaki, Kanagawa, Japan) [60]. The first fully expanded trifoliate leaf immediately below the flower bud was used for all leaf quality analyses on day 6 after vase solution treatment. After 20 min of dark adaptation, chlorophyll fluorescence was measured using a closed FluorCam (FC-800-C, PSI, Drásov, Czech Republic). The chlorophyll fluorescence parameters Fv/Fm, QY, NPQ, Rfd, and qP were determined. Fv/Fm (QY_max), expressed as Fv/Fm, represents the maximum quantum efficiency of PSII photochemistry. QY_Lss (Fq_Lss/Fm_Lss) represents the steady-state PSII quantum yield, whereas NPQ [(Fm − Fm_Lss)/Fm_Lss] represents steady-state non-photochemical quenching. Rfd [(Fp − Ft_Lss)/Ft_Lss] represents the fluorescence decline ratio in the steady state, and qP (Fq_Lss/Fv_Lss) represents the coefficient of photochemical quenching in the steady state [53]. The actinic light intensity was set at 1880 μmol·m^−2^·s^−1^, with a maximum measuring capacity of 4000 μmol·m^−2^·s^−1^. Chlorophyll fluorescence images were acquired using the Quenching Act 2 protocol, in which the shutter speed and camera sensitivity were automatically adjusted according to the background light intensity.

The rate of change in stomatal pore size was calculated as follows [61,62]:

Rate of change in pore size (%) = (Pore size after 12 h under dark conditions/Pore size after 12 h under light conditions) × 100

Total carbohydrate content was determined using the anthrone colorimetric method described by Kim et al. [63], with a modification in the absorbance measurement instrument. Briefly, oven-dried leaf samples were ground into a fine powder, and 0.05 g of the sample was hydrolyzed with 10 mL of 0.7 N HCl in a boiling water bath at 100 °C for 150 min. After cooling, the samples were centrifuged at 5000 rpm and 23 °C for 30 min. An aliquot (0.05 mL) of the supernatant was brought to a final volume of 1 mL with distilled water and mixed with 4 mL of 0.2% anthrone reagent prepared in 95% H_2_SO_4_. The mixture was heated at 100 °C for 8 min and subsequently cooled in an ice-water bath to terminate the reaction. Absorbance was measured at 620 nm using a microplate reader (SpectraMax ABS Plus Microplate Reader, Molecular Devices, LLC, San Jose, CA, USA) instead of the spectrophotometer used in the original method. Total carbohydrate content was quantified using a glucose standard curve.

### 4.3. Antimicrobial Effect

Bacterial populations in the vase solution were determined by collecting the vase solution containing the cut flower stems on day 4. The samples were serially diluted (10^3^–10^4^), and 1 mL aliquots of each dilution were plated in triplicate onto Petrifilm™ Aerobic Count Plates (3M Company, St. Paul, MN, USA) according to Park [57]. Plates were incubated at 35 °C for 48 h, and colonies were subsequently counted and expressed as colony-forming units per milliliter (CFU·mL^−1^).

### 4.4. Experimental Design and Data Analysis

The experiment was conducted using a completely randomized design. The number of replicates used for each measurement is indicated in the corresponding figures and tables. Data were analyzed using one-way analysis of variance (ANOVA), followed by Tukey’s honestly significant difference (HSD) test for multiple comparisons. Statistical analyses were performed using SAS software (Statistical Analysis System, version 9.4, SAS Institute Inc., Cary, NC, USA) and jamovi (version 2.7; The jamovi project). Differences were considered statistically significant at *p* < 0.05. To evaluate the interrelationships among the physiological and morphological parameters, Pearson’s correlation analysis was performed using the mean values for each treatment group. A correlation heatmap was generated using R (version 4.6.0; R Foundation for Statistical Computing, Vienna, Austria) with the ‘corrplot’ package. Hierarchical clustering was applied to the correlation matrix using the complete linkage method to identify clusters of variables with similar characteristics.

## 5. Conclusions

In conclusion, malic acid treatment resulted in the longest vase life and significantly extended vase life compared with distilled water in the cut rose ‘Candlelight’ grown under low-light conditions, whereas sucrose treatment shortened vase life despite increasing leaf carbohydrate content. Malic acid maintained higher vase solution uptake, relative fresh weight, and stem quality, whereas the combined application of malic acid and sucrose resulted in a significantly shorter vase life than malic acid alone. Additionally, vase life was positively associated with RFW and flower diameter and negatively associated with NPQ, Rfd, and the petal color parameters L* and C*. These results provide a basis for developing preservative solutions to improve the postharvest quality of cut roses produced under low-light conditions.

## 6. Patents

A patent application resulting from the work reported in this manuscript has been filed in the Republic of Korea: Korean Patent Application No. 10-2026-0190586, “Preservative Composition Comprising Malic Acid for Maintaining the Flower Color of Cut Roses, Preservation Method Using the Same, and Method for Preparing the Preservative Solution,” filed by the Jeju National University Industry-Academic Cooperation Foundation.

## Figures and Tables

**Table 1:** Symptoms associated with vase life termination in the cut rose ‘Candlelight’ treated with different vase solutions. The vase solutions consisted of distilled water (DW), tap water (TW), 1% chrysal (CR), 50 mg·L^−1^ malic acid (MA), 1% sucrose (Su), and 50 mg·L^−1^ malic acid + 1% sucrose (MA + Su).

Treatment	Petal Rolling	Wilting of Petal or Leaf	*Botrytis cinerea*	Petal Yellowing	Petal Malformation	Petal Abscission
DW	30	50	0	0	20	0
TW	0	60	0	30	20	0
CR	100	0	0	0	0	0
MA	60	50	0	10	0	0
Su	10	80	0	0	10	0
MA + Su	40	80	0	0	10	0

Values represent the percentage of cut flowers exhibiting each symptom at the end of vase life (*n* = 10). Individual cut flowers could exhibit more than one termination symptom; therefore, the percentages may not sum to 100%.

**Table 2:** The petal color and bacterial count in the vase solution of the cut rose ‘Candlelight’ treated with different vase solutions on day 4. The vase solutions consisted of distilled water (DW), tap water (TW), 1% chrysal (CR), 50 mg·L^−1^ malic acid (MA), 1% sucrose (Su), and 50 mg·L^−1^ malic acid + 1% sucrose (MA + Su).

Treatment	Petal Color				Bacterial Count (CFU · mL^−1^)
	L* ^z^	C* ^y^	h* ^x^	(ΔE* ^w^)	
DW	90.75 a ^v^	17.35 a	85.79 ab	11.87 a	5.50 × 10^5^ a
TW	87.57 a	15.45 a	84.37 ab	14.00 a	3.83 × 10^5^ a
CR	89.23 a	16.76 a	84.73 ab	13.05 a	ND ^u^ b
MA	88.66 a	16.31 a	78.64 ab	11.12 a	3.57 × 10^5^ a
Su	91.26 a	17.95 a	84.60 a	10.42 a	5.67 × 10^4^ b
MA + Su	89.57 a	17.47 a	86.96 b	11.28 a	3.77 × 10^5^ a

^z^ L*: Lightness (0 = black, 100 = white). ^y^ Chroma indicates the hypotenuse of a right triangle formed by joining points (0, 0), (a*, b*), and (a*, 0). ^x^ Hue indicates the angle between the hypotenuse and 0° on the a* axis. ^w^ ΔE* = [(ΔL)^2^ + (Δa)^2^ + (Δb)^2^]^1/2^. ^v^ Different lowercase letters within a column indicate significant differences among treatments according to Tukey’s honestly significant difference (HSD) test at *p* < 0.05. Sample sizes were *n* = 8–10 for petal color parameters and *n* = 3 for bacterial counts. ^u^ Not detected.

**Table 3:** Chlorophyll fluorescence (Fv/Fm), steady-state PSII quantum yield (QY_Lss), non-photochemical quenching (NPQ), fluorescence decline ratio (Rfd), and photochemical quenching coefficient (qP) of leaves of the cut rose ‘Candlelight’ treated with different vase solutions on day 4. The vase solutions consisted of distilled water (DW), tap water (TW), 1% chrysal (CR), 50 mg·L^−1^ malic acid (MA), 1% sucrose (Su), and 50 mg·L^−1^ malic acid + 1% sucrose (MA + Su).

Treatment	Chlorophyll Fluorescence (Fv/Fm)	QY_Lss	NPQ	Rfd	qP
DW	0.84 a ^z^	0.09 ab	2.03 a	2.28 a	0.15 ab
TW	0.82 a	0.07 b	2.02 a	2.39 a	0.11 b
CR	0.81 a	0.13 a	2.05 a	2.29 a	0.22 a
MA	0.85 a	0.09 b	2.17 a	2.42 a	0.14 ab
Su	0.83 a	0.09 b	3.26 a	3.69 a	0.18 ab
MA + Su	0.83 a	0.09 b	2.24 a	2.56 a	0.14 ab

^z^ Different lowercase letters within a column indicate significant differences among treatments according to Tukey’s honestly significant difference (HSD) test at *p* < 0.05 (*n* = 3).

## Data Availability

The original contributions presented in this study are included in the article. Further inquiries can be directed to the corresponding authors.
